# Effectiveness of COVID-19 vaccines in a large European hemodialysis cohort

**DOI:** 10.3389/fneph.2022.1037754

**Published:** 2022-11-15

**Authors:** Ana Paula Bernardo, Paola Carioni, Stefano Stuard, Peter Kotanko, Len A. Usvyat, Vratislava Kovarova, Otto Arkossy, Francesco Bellocchio, Antonio Tupputi, Federica Gervasoni, Anke Winter, Yan Zhang, Hanjie Zhang, Pedro Ponce, Luca Neri

**Affiliations:** ^1^ Fresenius Medical Care Portugal / Nephrocare Portugal, Vila Nova de Gaia, Portugal; ^2^ Unit for Multidisciplinary Research in Biomedicine (UMIB), Institute of Biomedical Sciences Abel Salazar (ICBAS), Porto University, Oporto, Portugal; ^3^ Fresenius Medical Care Italia SpA, Palazzo Pignano, Italy; ^4^ Fresenius Medical Care Deutschland GmbH, Bad Homburg, Germany; ^5^ Renal Research Institute, New York, NY, United States; ^6^ Icahn School of Medicine at Mount Sinai, New York, NY, United States; ^7^ Fresenius Medical Care, Waltham, MA, United States; ^8^ Fresenius Medical Care Portugal / Nephrocare Portugal, Lisboa, Portugal

**Keywords:** COVID-19, hemodialysis, SARS-CoV-2, mRNA vaccines, viral-carrier vaccines, effectiveness

## Abstract

**Background:**

Hemodialysis patients have high-risk of severe SARS-CoV-2 infection but were unrepresented in randomized controlled trials evaluating the safety and efficacy of COVID-19 vaccines. We estimated the real-world effectiveness of COVID-19 vaccines in a large international cohort of hemodialysis patients.

**Methods:**

In this historical, 1:1 matched cohort study, we included adult hemodialysis patients receiving treatment from December 1, 2020, to May 31, 2021. For each vaccinated patient, an unvaccinated control was selected among patients registered in the same country and attending a dialysis session around the first vaccination date. Matching was based on demographics, clinical characteristics, past COVID-19 infections and a risk score representing the local background risk of infection at vaccination dates. We estimated the effectiveness of mRNA and viral-carrier COVID-19 vaccines in preventing infection and mortality rates from a time-dependent Cox regression stratified by country.

**Results:**

In the effectiveness analysis concerning mRNA vaccines, we observed 850 SARS-CoV-2 infections and 201 COVID-19 related deaths among the 28110 patients during a mean follow up of 44 ± 40 days. In the effectiveness analysis concerning viral-carrier vaccines, we observed 297 SARS-CoV-2 infections and 64 COVID-19 related deaths among 12888 patients during a mean follow up of 48 ± 32 days. We observed 18.5/100-patient-year and 8.5/100-patient-year fewer infections and 5.4/100-patient-year and 5.2/100-patient-year fewer COVID-19 related deaths among patients vaccinated with mRNA and viral-carrier vaccines respectively, compared to matched unvaccinated controls. Estimated vaccine effectiveness at days 15, 30, 60 and 90 after the first dose of a mRNA vaccine was: for infection, 41.3%, 54.5%, 72.6% and 83.5% and, for death, 33.1%, 55.4%, 80.1% and 91.2%. Estimated vaccine effectiveness after the first dose of a viral-carrier vaccine was: for infection, 38.3% without increasing over time and, for death, 56.6%, 75.3%, 92.0% and 97.4%.

**Conclusion:**

In this large, real-world cohort of hemodialyzed patients, mRNA and viral-carrier COVID-19 vaccines were associated with reduced COVID-19 related mortality. Additionally, we observed a strong reduction of SARS-CoV-2 infection in hemodialysis patients receiving mRNA vaccines.

## 1 Introduction

A mass vaccination campaign against the severe acute respiratory syndrome coronavirus 2 (SARS-CoV-2) started worldwide since December 2020, using new vaccines that received the emergency use listing (EUL) from most of regulatory authorities. EUL was granted after proven efficacy in preventing laboratory-confirmed SARS-CoV-2 infection or symptomatic coronavirus disease 2019 (COVID-19), that varied between 50.7% and 95.0% in randomized controlled trials (1–6).

Patients receiving long-term hemodialysis (HD) treatment are particularly susceptible to SARS-CoV-2 infection (7–9) because self-isolation is not feasible in this cohort. End Stage Kidney Disease (ESKD) patients are also at high risk of more severe COVID-19 due to dysregulated immune functions (10–14) and high comorbidity burden (15, 16). As a result, the COVID-19 related mortality is as high as 32% among ESKD patients (17).

Although randomized controlled trials are considered the reference standard for evaluating quality, safety and efficacy of COVID-19 vaccines, they are limited by sample size and lack of representation of specific high-risk groups, including patients with ESKD receiving dialysis (1–6). These data are critical and may influence clinical practice, as hemodialysis patients showed a reduced antibody response to mRNA vaccines when compared with healthy controls (18–22).

The extent to which humoral response contributes to COVID-19 vaccine efficacy is unknown, and there are not yet universally validated and accepted antibody cutoffs that correlate with protection against severe COVID-19 courses in patients under dialysis (19).

For these reasons, a study evaluating real-world effectiveness of COVID-19 vaccines in ESKD patients undergoing dialysis is needed.

In this study, we evaluated the effectiveness of COVID-19 vaccines against documented SARS-CoV-2 infection and COVID-19 related death in patients receiving in-center hemodialysis therapy in Fresenius Medical Care (FMC) Nephrocare (NC) European centers with available data in a noncontrolled setting.

## 2 Materials and methods

### 2.1 Study design, patients, and setting

In this historical 1:1 matched cohort study, we included all adult individuals receiving in-center hemodialysis therapy in FMC NC European dialysis centers in the period from December 1, 2020, to May 31, 2021 (study period) who granted permission to use their pseudo-anonymized data for secondary data analysis. All patients’ data have been extracted from the European Clinical Database (EuCliD^®^, Fresenius Medical Care, Deutschland GmbH, Vaiano Cremasco, Italy) (23–26), the health information system adopted by more than 1000 FMC dialysis centers in 43 countries worldwide.

Eligibility criteria included an age of 18 years or older, having dialysis treatments recorded in the 14 days prior to index date and after index date, renal replacement therapy onset date available, not having a documented SARS-CoV-2 infection within the last 30 days prior to the index date. We excluded all patients from countries that did not systematically report COVID-19 cases in EuCliD^®^, namely, the United Kingdom, Ireland, Israel, Lebanon, and Switzerland.

### 2.2 Endpoint definition

The outcomes of interest were documented SARS-CoV-2 infection, defined by the presence of at least one nasopharyngeal swab (or respiratory sample, if the patient was hospitalized) that was positive for SARS-CoV-2 by reverse-transcriptase-polymerase-chain-reaction (RT-PCR) test, and COVID-19 related death, defined as a death occurred after a documented SARS-CoV-2 infection in the study period. Since April 2020, suspected and confirmed SARS-CoV-2 infections have been tracked in the Treatment Incident Reporting (TIR) module in EuCliD^®^ with the initial symptoms, diagnostic tests and clinical outcomes, allowing to promptly detect the spread of the disease in the individual dialysis centers and to act promptly on the proximal units, in an efficient and reliable way.

### 2.3 Exposure groups

We contrasted outcomes occurrence between vaccinated and unvaccinated patients against SARS-CoV-2 infection. For each matched person included in the study, follow up ended at the earliest of the following events: occurrence of an outcome event (SARS-CoV-2 infection or COVID-19 related death), vaccination (for unvaccinated cohort), vaccination of the matched control (for vaccinated cohort), or the end of the study period. Vaccinated persons censored due to censoring of their matched unvaccinated control are definitively censored and not re-included in the vaccination cohort for a new matching. Newly vaccinated persons are eligible for inclusion in the study in the vaccination cohort, even if they had previously been selected as a control. In this last case, the follow up period related to the control status ends the day before the vaccination date.

### 2.4 Covariates

As detailed in **Supplementary Table S1**, covariates at patient level considered for all statistical approaches included country, demographics characteristics, etiology, comorbidities, past SARS-CoV-2 infections, dialysis related parameters, laboratory serum levels, medications, history of hospitalizations and were calculated/collected over different timeframes in the 6 months prior to the index date (ascertainment period).

### 2.5 Statistical analysis

We computed mean and standard deviation or absolute and relative frequencies for continuous or discrete variables respectively. Differences in patients’ characteristics across study groups were compared with the student’s t test and the Pearson’s chi-squared test where appropriate. We computed incidence densities and 95% confidence intervals based on the Poisson distribution. P-values<0.05 denoted statistical significance. We conducted all analyses with SAS 9.4^®^.

#### 2.5.1 Matching strategy

To reduce selection bias due to nonrandomized vaccination allocation, we matched vaccinated patients in a one-to-one ratio to unvaccinated controls treated in the same country. Each day during the study period, for each newly vaccinated patient, we identified a group of eligible control patients among unvaccinated patients registered in the same country who received a dialysis session within +/- 3 days of the vaccination date. The index date was the date of the first vaccination for the vaccinated patient and the matching treatment date for the unvaccinated controls. Among the identified set of potential controls for each vaccinated patient (exact matching on country and index date), we performed an additional selection step based on a probabilistic matching approach. To ensure that patients included in the two exposure groups (vaccinated vs unvaccinated) would face the same background infection risk at study entry, matching was based on a time-varying outcome risk score (ORS) representing the likelihood of SARS-CoV-2 infection in the 14 days after the index date given local background risk of SARS-CoV-2 infection at index date as well as demographics and clinical characteristics collected in the ascertainment period.

Each day during the study period, we used the greedy nearest neighbor matching algorithm (27). After sorting the vaccinated patients in random order of the outcome risk score, the first vaccinated patient is matched to the control individual with the minimal outcome risk score difference. In this 1:1 matching, once two patients have been matched, they are removed from the set of subjects available for subsequent matching that proceed in the random order of the vaccinated patients until it is not possible to make more matching. We restricted the matching among those observations whose scores lied in the region of common support for the vaccinated and the control groups. The region of common support is the largest interval that contains scores for subjects in both groups: the lower limit is the largest of the minimum scores for the two groups, and the upper limit is lowest of the maximum scores for the two groups. We explored matching with different calipers of the outcome risk score to balance the trade-off between patients’ similarity within pairs and sample size (**Supplementary Table S2**). The final caliper used was 0.1.

We evaluated the covariate balance after matching by examining the effect size of the difference in clinical parameters and predicted infection rates across the matched samples. We used Cohen’s d for continuous variables (28) and Cramér’s V for categorical ones (29) considering acceptable difference of 0.25 or less (27, 30, 31). A negative value of the effect size means that the incidence in the unvaccinated cohort is lower than the incidence in the vaccinated cohort.

#### 2.5.2 Estimation of the outcome risk score

We estimated the parameters of the time-varying outcome risk score model using a stepwise logistic regression model (with significance threshold of P-value less than 0.30 for variable entry and 0.15 for variable removal) predicting the SARS-CoV-2 infection in the 14 days after the index date. We trained the ORS model on unvaccinated patients satisfying the same eligibility criteria of the study eligible controls as they were selected to match index dates every 1^st^ and 15^th^ day in the period from August 1, 2020, to November 30, 2020 (training dataset). The covariates used in the model are detailed in **Supplementary Table S1**. Besides socio-demographic and clinical characteristics, the ORS model also included the local (dialysis center) background risk of SARS-CoV-2 infection at index date. The local (dialysis center) background risk of SARS-CoV-2 infection was estimated by an enhanced sentinel surveillance system based on Artificial Intelligence (AI) (32). This sentinel surveillance system exploits the interconnection of the FMC NC European centers to estimate the risk of a COVID-19 outbreak in each dialysis center within a 2-week prediction horizon. Inputs of the model included open-source regional epidemic metrics and the accurately reported epidemic dynamics in each dialysis unit, propagated through distance-weighted metrics to the adjacent interconnected dialysis units, as well as the trends in clinical practice patterns. This AI model accurately captures the baseline local risk of SARS-CoV-2 infection associated to each clinic where the patients were treated at their index date: the area under the curve (AUC) of the receiver operating characteristics (ROC) curve was 0.81.

As detailed in **Supplementary Table S3**, we applied data cleansing to 23 continuous variables considering as missing any data that lied outside the listed upper or lower values. The **Table S3** also shows the amount of missing data after cleansing procedure. We input missing values, resulting from the above-mentioned data cleansing procedure or native in the data, with the national average calculated on the extracted original dataset (i.e., vaccinated and eligible controls). Before matching, eligible control patients are duplicated on each vaccination date in which they were eligible.

The outcome risk score model performance was evaluated measuring the AUC-ROC curve for the training dataset (unvaccinated patients treated in the period from August 1, 2020, to November 30, 2020), for the testing dataset (patients eligible to be included in the unvaccinated cohort of the study period), and for the dataset including patients eligible to be included in the vaccinated and unvaccinated cohorts of the study period.

#### 2.5.3 Estimation of vaccine effectiveness

We modelled the risk of documented SARS-CoV-2 infection and COVID-19 related death with extended Cox Regression models accounting for non-proportional hazard (where required) stratified by country and using a robust sandwich covariance matrix estimate to account for the matched-pair dependence. We performed two separate analyses, one for each vaccine type (mRNA or viral-carrier). We estimated the effectiveness of the COVID-19 vaccines in preventing SARS-CoV-2 infections and death as 1 minus the hazard ratio.

## 3 Results

### 3.1 Sample characteristics

**Figure 1:**
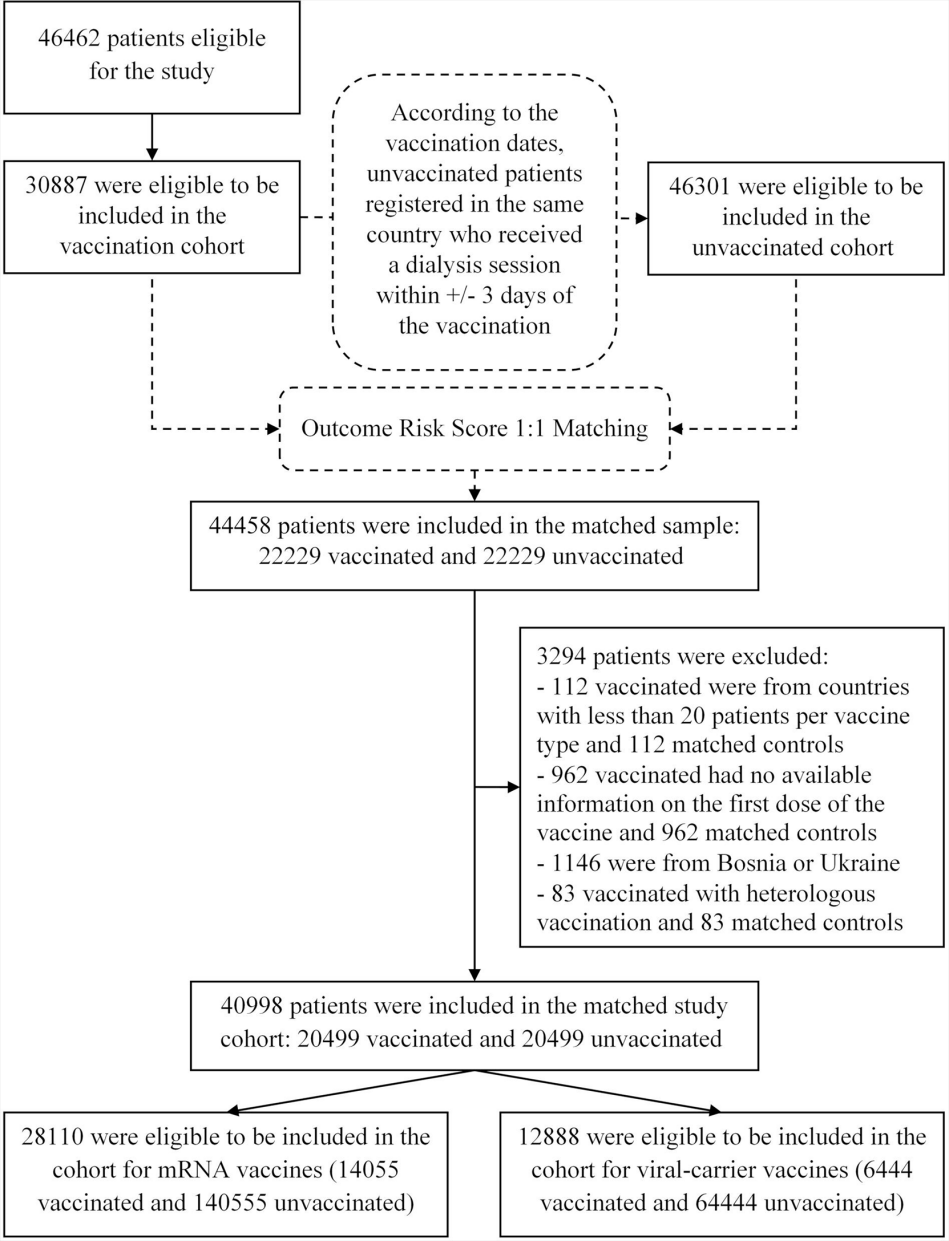
Study Population and Cohort Enrollment.

Among the 46462 patients treated in 539 dialysis centers located in 17 countries of the FMC NC European network eligible for the study, 30887 vaccinated patients were eligible to be included in the vaccination cohort. Concurrently, 46301 were eligible to be included in the unvaccinated cohort (**Figure 1**). The matched sample included 44458 patients (22229 vaccinated and 22229 unvaccinated). We additionally excluded patients vaccinated with missing information on the first dose of the vaccine, patients with heterologous vaccination, patients treated in countries with less than 20 patients per vaccine type and from Bosnia and Ukraine because the follow up was too short. The final matched study cohort included 40998 patients (20499 vaccinated and 20499 unvaccinated) with 6757 patients who switched their exposure status during the study period. These patients were treated in 524 dialysis centers located in 15 countries. The disposition of vaccinated patients in the matched study cohort, by country and type of vaccine is provided in **Supplementary Table S4**.

**Figure 2:**
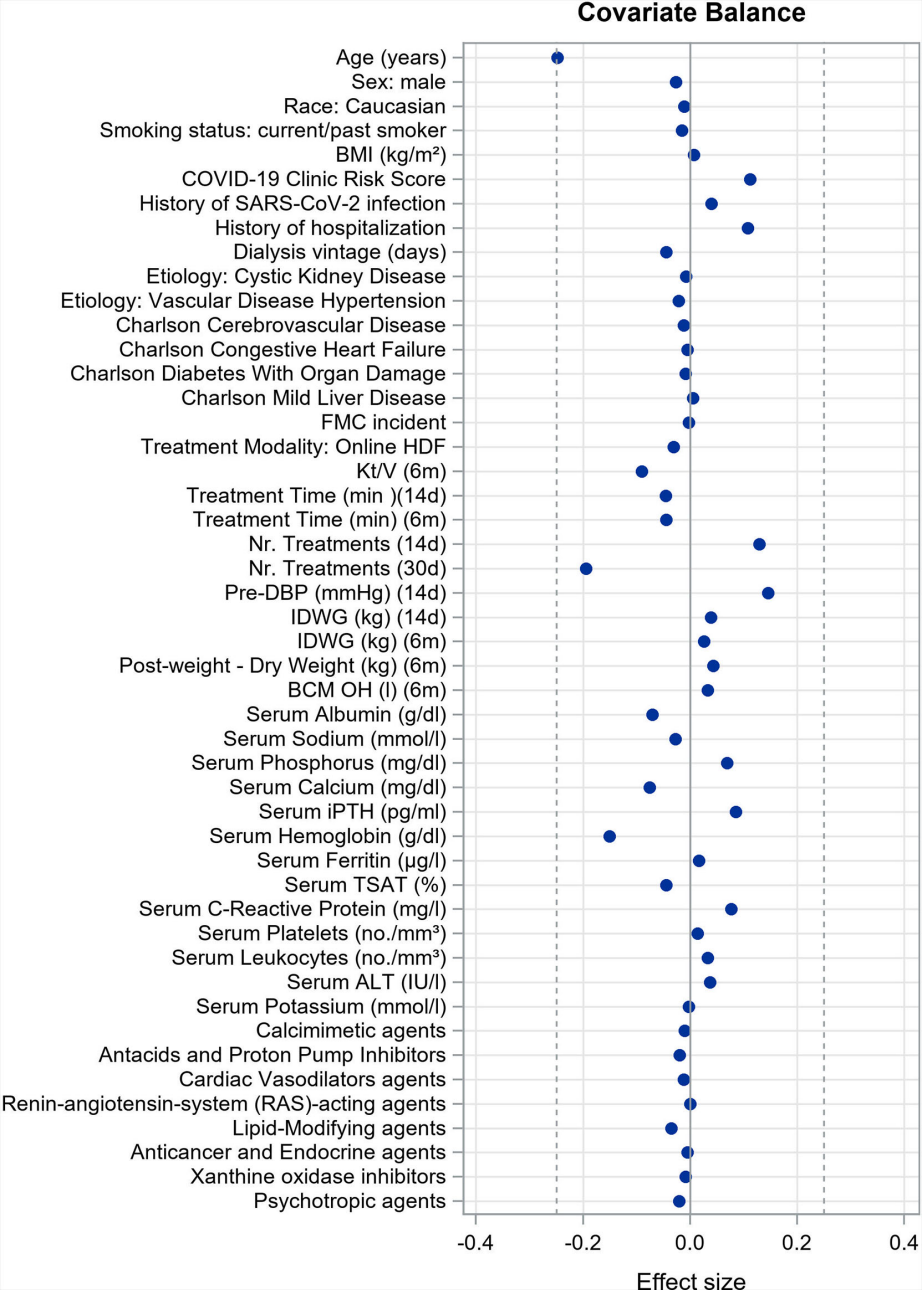
Covariate balance in matched study cohort showing the mean differences (unvaccinated group minus vaccinated group) for the significant covariates of the outcome risk score model. We used Cohen’s d for continuous variables and Cramér’s V for categorical ones considering acceptable difference of 0.25 or less.

Caliper matching through outcome risk score allowed a large common support region between vaccinated and unvaccinated cohorts, meaning the baseline risk of infection for the two cohorts was extremely overlapping. This is reflected in minimal imbalance in covariate distribution between cohorts after matching as reflected by very small effect size estimates for each comparison (**Figure 2**). Demographic and clinical characteristics of vaccinated persons and unvaccinated controls at baseline of the matched study sample are described in **Table 1**. Covariate balance distribution between cohorts and demographic and clinical characteristics of vaccinated persons and unvaccinated controls at baseline in the sample for mRNA vaccines is described in **Supplementary Figure S1** and **Supplementary Table S5**, while for viral-carrier vaccines in **Supplementary Figure S2** and **Supplementary Table S6**.

**Table 1:** Demographic and clinical characteristics of vaccinated persons and unvaccinated controls at baseline of the matched sample (20499 vaccinated and 20499 unvaccinated).

Characteristics	Unvaccinated(N=20499)	Vaccinated(N=20499)	Effect Size	Mean Difference [95% CI]
Age (years)	62.8 ± 14.6	66.3 ± 13.8	-0.248	-3.523 [-3.798, -3.249]
Sex: male	58.3	60.9	-0.027	-0.026 [-0.036, -0.017]
Race: Caucasian	57.3	58.3	-0.011	-0.011 [-0.020, -0.001]
Smoking status: current/past smoker	22.7	24	-0.015	-0.013 [-0.021, -0.005]
BMI (kg/m²)	27.5 ± 5.8	27.4 ± 5.5	0.007	0.038 [-0.071, 0.147]
Local (dialysis center) background risk of SARS-CoV-2 infection (32)	0.4 ± 0.4	0.4 ± 0.4	0.112	0.041 [0.034, 0.048]
History of SARS-CoV-2 infection	20.7	17.5	0.040	0.031 [0.024, 0.039]
History of hospitalization	0.4 ± 0.8	0.3 ± 0.7	0.108	0.080 [0.066, 0.095]
Dialysis vintage (days)	1870.1 ± 1723.9	1946.6 ± 1686.0	-0.045	-76.508 [-109.518, -43.497]
Etiology: Cystic Kidney Disease	6.4	6.7	-0.007	-0.004 [-0.008, 0.001]
Etiology: Vascular Disease Hypertension	10.5	11.8	-0.021	-0.013 [-0.019, -0.007]
Charlson Cerebrovascular Disease	14	14.8	-0.012	-0.008 [-0.015, -0.001]
Charlson Congestive Heart Failure	24.5	24.9	-0.005	-0.004 [-0.013, 0.004]
Charlson Diabetes with Organ Damage	26.8	27.6	-0.009	-0.008 [-0.016, 0.001]
Charlson Mild Liver Disease	11.5	11.1	0.005	0.003 [-0.003, 0.009]
FMC incident	78.5	78.6	-0.002	-0.002 [-0.010, 0.006]
Treatment Modality: Online HDF	52	55	-0.031	-0.031 [-0.040, -0.021]
Kt/V (6m)	1.6 ± 0.3	1.7 ± 0.3	-0.090	-0.031 [-0.037, -0.024]
Treatment Time (min) (14d)	244.4 ± 32.5	245.7 ± 27.9	-0.045	-1.375 [-1.961, -0.788]
Treatment Time (min) (6m)	243.5 ± 32.0	244.8 ± 26.9	-0.045	-1.321 [-1.894, -0.749]
Nr. Treatments (14d)	6.6 ± 1.3	6.4 ± 0.8	0.129	0.142 [0.120, 0.163]
Nr. Treatments (30d)	12.6 ± 2.9	13.1 ± 1.6	-0.194	-0.463 [-0.509, -0.417]
Pre-DBP (mmHg) (14d)	72.9 ± 12.3	71.2 ± 12.0	0.146	1.769 [1.534, 2.004]
IDWG (kg) (14d)	2.2 ± 1.1	2.1 ± 1.0	0.039	0.042 [0.021, 0.062]
IDWG (kg) (6m)	2.1 ± 0.9	2.1 ± 0.8	0.026	0.022 [0.006, 0.038]
Post-weight - Dry Weight (kg) (6m)	0.3 ± 0.6	0.3 ± 0.6	0.043	0.026 [0.014, 0.037]
BCM OH (l) (6m)	1.8 ± 1.4	1.7 ± 1.4	0.033	0.045 [0.019, 0.072]
Serum Albumin (g/dl)	3.9 ± 0.4	3.9 ± 0.4	-0.070	-0.026 [-0.034, -0.019]
Serum Sodium (mmol/l)	138.3 ± 3.1	138.4 ± 3.1	-0.027	-0.084 [-0.144, -0.024]
Serum Phosphorus (mg/dl)	4.7 ± 1.3	4.6 ± 1.3	0.070	0.090 [0.065, 0.115]
Serum Calcium (mg/dl)	8.8 ± 0.7	8.9 ± 0.7	-0.075	-0.051 [-0.064, -0.038]
Serum iPTH (pg/ml)	370.2 ± 317.7	343.9 ± 298.4	0.085	26.272 [20.305, 32.239]
Serum Hemoglobin (g/dl)	11.1 ± 1.4	11.3 ± 1.3	-0.151	-0.201 [-0.227, -0.175]
Serum Ferritin (µg/l)	616.6 ± 455.1	609.2 ± 443.6	0.017	7.436 [-1.264, 16.136]
Serum TSAT (%)	31.2 ± 16.0	31.9 ± 16.1	-0.044	-0.714 [-1.025, -0.403]
Serum C-Reactive Protein (mg/l)	11.7 ± 14.9	10.6 ± 13.5	0.077	1.095 [0.819, 1.371]
Serum Platelets (no./mm³)	195152.0 ±70206.9	194182.6 ± 66263.1	0.014	969.416 [-352.178, 2291.009]
Serum Leukocytes (no./mm³)	6687.4 ± 2073.9	6620.5 ± 1992.1	0.033	66.919 [27.552, 106.285]
Serum ALT (IU/l)	14.9 ± 8.5	14.6 ± 8.2	0.038	0.316 [0.153, 0.478]
Serum Potassium (mmol/l)	4.9 ± 0.7	4.9 ± 0.7	-0.003	-0.002 [-0.016, 0.012]
Calcimimetic agents	10.4	11	-0.010	-0.006 [-0.012, -0.000]
Antacids and Proton Pump Inhibitors	40.6	42.6	-0.020	-0.020 [-0.029, -0.010]
Cardiac Vasodilators agents	6.6	7.2	-0.012	-0.006 [-0.011, -0.001]
Renin-angiotensin-system (RAS)-acting agents	26.7	26.7	0.000	0.000 [-0.008, 0.009]
Lipid-Modifying agents	29	32.2	-0.035	-0.032 [-0.041, -0.023]
Anticancer and Endocrine agents	1	1.1	-0.005	-0.001 [-0.003, 0.001]
Xanthine oxidase inhibitors	15.4	16	-0.008	-0.006 [-0.013, 0.001]
Psychotropic agents	22.5	24.2	-0.020	-0.017 [-0.025, -0.009]

The 6757 patients who switched their exposure status during the study period appear in both groups. Continuous variables are expressed as mean ± standard deviation, categorical variables are expressed as percentage. The mean differences and 95% confidence interval (CI) are related to unvaccinated group minus vaccinated group. For effect size, we used Cohen’s d for continuous variables and Cramér’s V for categorical ones considering acceptable difference of 0.25 or less. BMI, body mass index. FMC incident, if the patient started the renal replacement therapy not more than 3 months before FMC admission. Online HDF, Online hemodiafiltration. DBP, diastolic blood pressure. IDWG, interdialytic weight gain. BCM OH, overhydration by the body composition monitor (BCM; Fresenius). TSAT, transferrin saturation. ALT, alanine transaminase. 6m, 6 months prior to index date. 14d, past 14 days prior to index date. 30d, past 30 days prior to index date.

### 3.2 Effectiveness of mRNA vaccines

**Figure 3:**
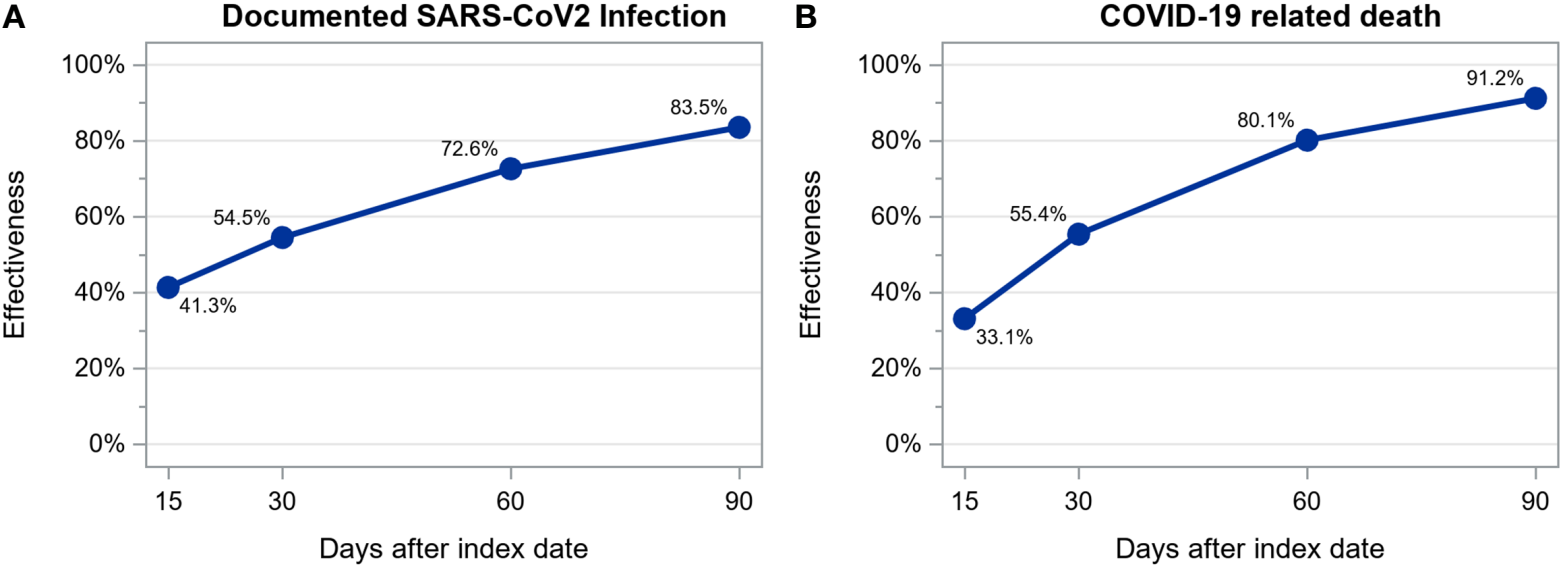
Estimation of mRNA vaccines effectiveness in preventing documented SARS-CoV-2 infection **(A)** and COVID-19 related death **(B)** calculated as 1 – hazard ratio estimated from a time-dependent extended Cox regression stratified by country.

We observed 850 SARS-CoV-2 infections among 28110 patients (14055 vaccinated and 14055 unvaccinated, treated in 421 dialysis centers located in 14 countries) during a mean follow up time of 44 ± 40 days: 562 (35.6/100 person-years; 95% CI: 32.7-38.6/100 person-years) among unvaccinated and 288 (17.1/100 person-years; 95% CI: 15.2-19.2/100 person-years) among vaccinated patients. Of these 850 infections, 201 resulted in death: 145 (8.7/100 person-years; 95% CI: 7.4-10.2/100 person-years) among unvaccinated and 56 (3.3/100 person-years; 95% CI: 2.5-4.2/100 person-years) among vaccinated patients. Estimated vaccine effectiveness at days 15, 30, 60 and 90 after the first dose of a mRNA vaccine was as follows: for infection, 41.3%, 54.5%, 72.6% and 83.5% and, for death, 33.1%, 55.4%, 80.1% and 91.2% (**Figure 3**). The number of patients at risk at each time point and the cumulative number of events for each outcome are shown in **Supplementary Table S7**.

### 3.3 Effectiveness of viral-carrier vaccines

**Figure 4:**
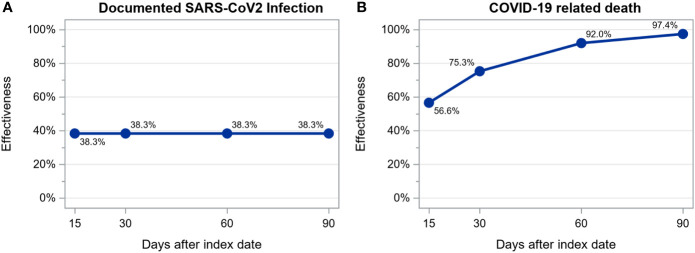
Estimation of viral-carrier vaccines effectiveness in preventing documented SARS-CoV-2 infection **(A)** and COVID-19 related death **(B)** calculated as 1 – hazard ratio estimated from a time-dependent extended Cox regression stratified by country.

We observed 297 SARS-CoV-2 infections among 12888 patients (6444 vaccinated and 6444 unvaccinated, treated in 223 dialysis centers located in 7 countries) during a mean follow up time of 48 ± 32 days: 181 (22.1/100 person-years; 95% CI: 19.1-25.5/100 person-years) among unvaccinated and 116 (13.6/100 person-years; 95% CI: 11.4-16.3/100 person-years) among vaccinated patients. Of these 297 infections, 64 resulted in death: 54 (6.4/100 person-years; 95% CI: 4.9-8.4/100 person-years) among unvaccinated and 10 (1.2/100 person-years; 95% CI: 0.6-2.2/100 person-years) among vaccinated patients. Estimated vaccine effectiveness at days 15, 30, 60 and 90 after the first dose of a viral-carrier vaccine was as follows: for infection, 38.3% without any increase over time and, for death, 56.6%, 75.3%, 92.0% and 97.4% (**Figure 4**). The number of patients at risk at each time point and the cumulative number of events for each outcome are shown in **Supplementary Table S8**.

## 4 Discussion

This retrospective observational study provides real-world estimates of high effectiveness of mRNA vaccines and viral carrier vaccines against COVID-19 related mortality, and also high effectiveness of mRNA vaccines against SARS-CoV-2 infection in ESKD patient receiving in-center hemodialysis.

In the cohort of patients analyzed to estimate the effectiveness of mRNA vaccines in preventing SARS-CoV-2 infection, a total of 850 infections were observed, with a significant lower incidence density among vaccinated patients (17.1/100 person-years) compared to unvaccinated (35.6/100 person-years). We also observed a significant lower incidence density of SARS-CoV-2 infections among patients vaccinated with viral-carrier vaccines (13.6/100 person-years) compared to the control group of unvaccinated patients (22.1/100 person-years).

Several studies demonstrated reduced antibody responses of dialyzed patients to mRNA vaccines compared to healthy controls (18–22). Consistent with previous case-control and historical cohort studies (33–36), our results demonstrated, high clinical effectiveness of both mRNA and viral carrier vaccines against COVID-19 related mortality in a large real-world hemodialysis patient cohort, a high-risk population that frequently suffer from other medical conditions that can compromise antibody response beyond the impaired immune response that is observed in ESKD *per se* (10, 12–14). These observations may have important clinical implications for clinical practice.

Our results show that the mortality risk associated with SARS-CoV-2 infection was lower in patients vaccinated with mRNA vaccines compared to unvaccinated controls (3.3 vs 8.7/100 person-year). The same was observed concerning viral-carrier vaccines, with a lower mortality risk observed in patients vaccinated with viral-carrier vaccines compared to unvaccinated controls (1.2 vs 6.4/100 person-years). These real-world results show the importance of careful interpretation of existing reports indicating a highly diminished antibody response after COVID-19 vaccination in ESKD patients compared with healthy controls (18–22). This is supported by the fact that there are not universally validated and accepted antibody cutoffs correlated with protection against severe COVID-19 courses. The extent to which humoral response contributes to vaccinal protection in COVID-19 is unknown as well. Our study adds insights into the hypothesis of cellular immune response contribution on protection against infection or reduction of severe COVID-19 disease, even in dialysis patients with a lower humoral response to COVID-19 vaccines.

We observed an increase in effectiveness of both mRNA and viral-carrier vaccines against COVID-19 related mortality over time, at least in the first 90 days after the first dose. This observation was expected as the index date was coincident with the date of vaccine first dose administration and most vaccines evaluated in our study were administered as a two-dose regimen (1, 37). The cumulative number of patients with two doses administered in their follow-up period is shown in **Supplementary Table S9**. On the contrary, the interaction term denoting the change in effectiveness along time for viral-carrier vaccines in reducing infection rates was not statistically significant; the interpretation of such result is not straightforward as lack of statistical significance does not necessarily mean lack of effect; in fact, our study may be insufficiently powered to detect small interaction effects, despite present.

Our study has several methodological strengths, the first being the large number of patients evaluated. We also performed a pair matched analysis for 88 variables that could have influenced the patient outcomes, including background epidemic risk of infection, socio-demographic factors as well as comorbidities, dialysis related parameters, and biochemical markers serum concentrations. The outcome risk score model incorporated in our matching strategy (32) allowed a precise risk assessment for a COVID-19 outbreak in our dialysis clinics over a 2-week forecasting horizon and therefore allows to effectively balance the risk of infection in the matched cohorts of vaccinated and unvaccinated patients, because this model captures the local disease spread in the particular and high-risk setting, the dialysis unit, where the human interactions are numerous and unavoidable, more precisely than the simple epidemic status in the general population. The accuracy of our sentinel surveillance system is ensured by our epidemic tracing procedure (e.g. Treatment Incident Reporting) which allows to promptly record any SARS-CoV-2 infections in each individual dialysis centers belonging to the network. The enhanced sentinel surveillance system had a stable accuracy over time and was able to consistently discriminate outbreak risk in FMC NC dialysis units across all European countries and, for that reason, we can assure that both the vaccinated patients and the unvaccinated controls were exposed at the same risk for SARS-CoV-2 infection at study entry. During the study period, there were no differences within each country, between vaccinated and unvaccinated persons concerning different testing behaviors or levels of adherence to non-pharmacologic interventions which might have confounded our vaccine effectiveness estimates. Finally, misclassification of vaccine history in our study is unlikely because of comprehensive recording of vaccine administration in our dialysis units.

Our study has some limitations, the most important being the short follow-up. Longer-term data on effectiveness are needed, especially in an environment where new SARS-CoV-2 variants continue to emerge. Another limitation is that the time from symptom onset to death might have precluded the identification of all COVID-19 related deaths during the study period. However, this might have occurred in both sub-groups of vaccinated and unvaccinated patients, and a differential effect between the two sub-groups is not expected. Finally, we would like to emphasize that these results can only be applied both to the D614G SARS-CoV-2 variant and for the B.1.17 variant, as these were the two dominant variants in the European countries, where this study was performed, during the study period (between December 2020 and May 2021).

Our study assessed the real-word clinical effectiveness of COVID-19 vaccines in a large number of patients under hemodialysis, and it demonstrates that COVID-19 vaccines approved from most of regulatory authorities are effective in reducing COVID-19 related mortality (at least with variants D614G and B.1.17) in this high-risk population. A strong reduction in SARS-CoV-2 infection was also observed in dialysis patients receiving an mRNA vaccine, while viral-carrier vaccines were less protective against infection. It is reassuring that the vaccination campaign showed to provide protection in this understudied population despite health authorities had to take hard decisions in an emergency situation in which dialysis patients were not represented in randomized clinical trials for COVID-19 vaccines.

Whether this lesson is applicable for a different pandemic scenario with a different vaccine and pathogen is difficult to evaluate. In the event of an outbreak of COVID-19 variants, it seems reasonable to apply a similar vaccination policy for dialysis patients and the general population. However, given the lack of randomized clinical trials, it would be key to closely monitor the effectiveness and safety of new vaccine formulations in dialysis patients. Our data showed that a shared system for reporting and tracking suspected and confirmed infected cases, such as the Treatment Incident Reporting System, which connects all operating units within FMC NC European network, was key to understand the epidemic dynamics in the community, to evaluate the effectiveness of pharmacological and non-pharmacological intervention and to implement enhanced sentinel surveillance systems for population health governance.

Understanding the clinical impact of mRNA vaccines against SARS-CoV-2 variants and related mortality in hemodialysis patients is warranted.

## Data availability statement

The datasets presented in this article are not readily available because they include protected health information. Specific, well-motivated, requests to access the pseudo-anonymized analytic dataset may be considered by the authors. Requests to access the datasets should be directed to LN, Luca.Neri@fmc-ag.com.

## Ethics statement

The studies involving human participants were reviewed and approved by Institutional Review Board of FMC-Nephrocare Portugal. The patients/participants provided their written informed consent for secondary analysis of their data. Written informed consent for participation was not required for this study in accordance with the national legislation and the institutional requirements.

## Author contributions

Conceptualization: LN, SS, PK, LU, AW, VK, OA. Data curation: PC and AT. Formal analysis: PC. Supervision and validation: LN, SS and FB. Writing—original draft: PC and AB. Interpretation of results, writing—review and editing: All authors. All authors contributed to the article and approved the submitted version.

## Acknowledgments

We would like to thank John W. Larkin for his substantial contribution to the conceptualization of our study. Also, we would like to thank the patient care teams at Fresenius Medical Care who captured the data used in this analysis during the provision of standard medical care.

## Conflict of interest

AB, PC, SS, LU, VK, OA, FB, AT, FG, AW, YZ, PP and LN are employees of Fresenius Medical Care. PK and HZ are employees of the Renal Research Institute, a wholly owned subsidiary of Fresenius Medical Care. LU and PK have share options/ownership in Fresenius Medical Care. PK, HZ, LU are inventors on patents in the field of dialysis. PK receives honorarium from UpToDate and HS Talks, and is on the Editorial Board of Blood Purification, Frontiers in Nephrology, Kidney and Dialysis, and Kidney and Blood Pressure Research.

## Publisher’s note

All claims expressed in this article are solely those of the authors and do not necessarily represent those of their affiliated organizations, or those of the publisher, the editors and the reviewers. Any product that may be evaluated in this article, or claim that may be made by its manufacturer, is not guaranteed or endorsed by the publisher.
