# Supplementary material for: Effectiveness of COVID-19 vaccines in a large European hemodialysis cohort
Source: Front Nephrol. 2022 Nov 15;2:1037754. doi: 10.3389/fneph.2022.1037754 (PMC10479614; doi:10.3389/fneph.2022.1037754)
Supplement: Supplementary file 1 [file DataSheet_1.pdf]

Supplementary Material

Contents

1 Supplementary Figure S1 - Covariate balance distribution in the mRNA vaccines cohort 2
2 Supplementary Figure S2 - Covariate balance distribution in the viral-carrier vaccines cohort 3
3 Supplementary Table S1 – Covariates included in the outcome risk score model 4
4 Supplementary Table S2 – Caliper matching 8
5 Supplementary Table S3 – Data management 9
6 Supplementary Table S4 – Vaccinated patients by country and vaccine type 10
7 Supplementary Table S5 - Baseline characteristics of the mRNA vaccines cohort 11
8 Supplementary Table S6 - Baseline characteristics of the viral-carrier vaccines cohort 14
9 Supplementary Table S7 – No. At Risk and Events in the mRNA vaccines cohort 17
10 Supplementary Table S8 – No. At Risk and Events in the viral-carrier vaccines cohort 18
11 Supplementary Table S9 – Cumulative patients with 2 doses of COVID-19 vaccine 19
12 References 20

1 Supplementary Figure S1 - Covariate balance distribution in the mRNA vaccines cohort

Figure S1. Covariate balance distribution in the sample for mRNA vaccines showing the mean differences (unvaccinated group minus vaccinated group) for the significant covariates of the outcome risk score model. We used Cohen's d for continuous variables and Cramér's V for categorical ones considering acceptable difference of 0.25 or less.

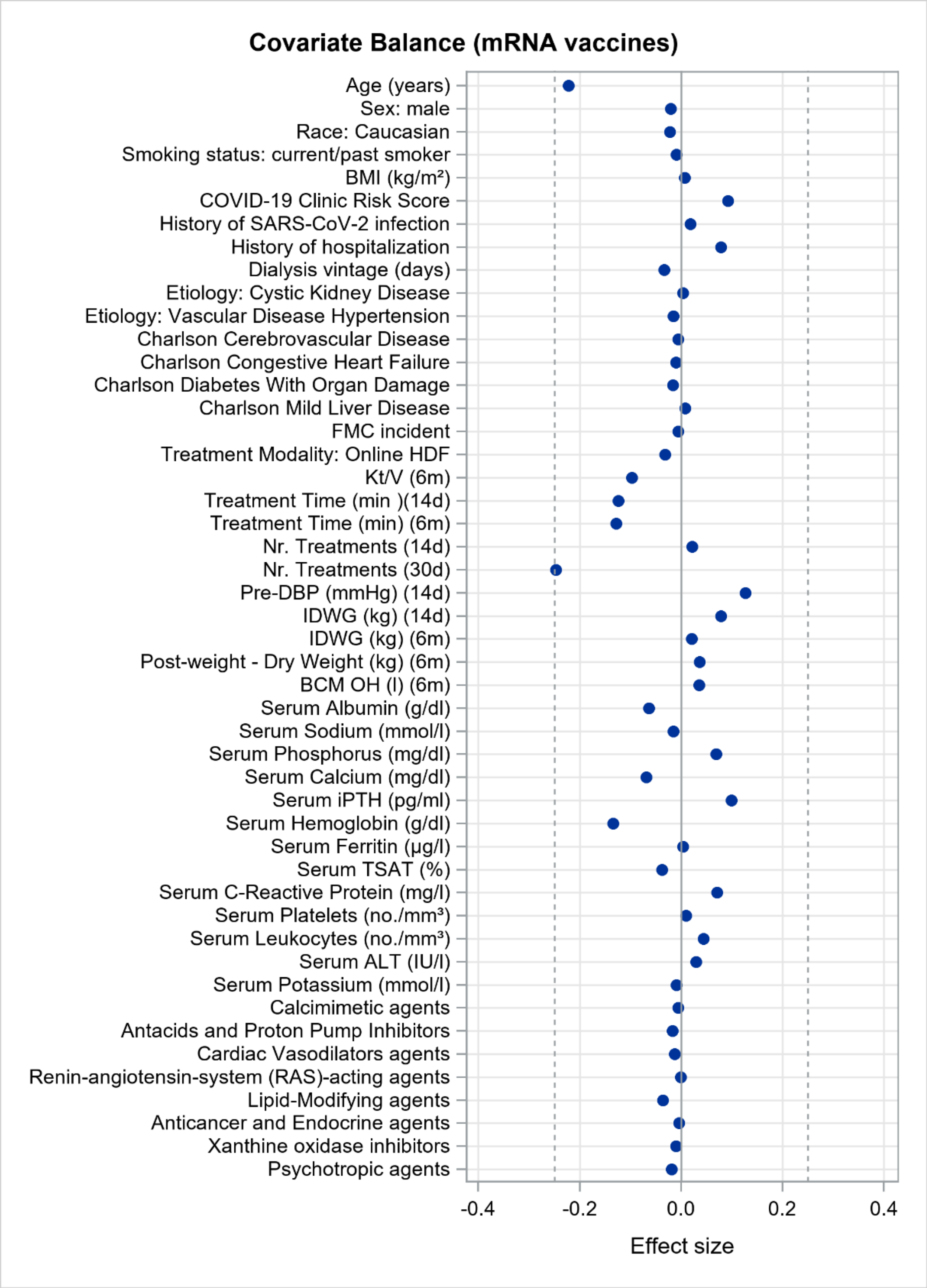

2 Supplementary Figure S2 - Covariate balance distribution in the viral-carrier vaccines cohort

Figure S2. Covariate balance distribution in the sample for viral-carrier vaccines showing the mean differences (unvaccinated group minus vaccinated group) for the significant covariates of the outcome risk score model. We used Cohen's d for continuous variables and Cramér's V for categorical ones considering acceptable difference of 0.25 or less.

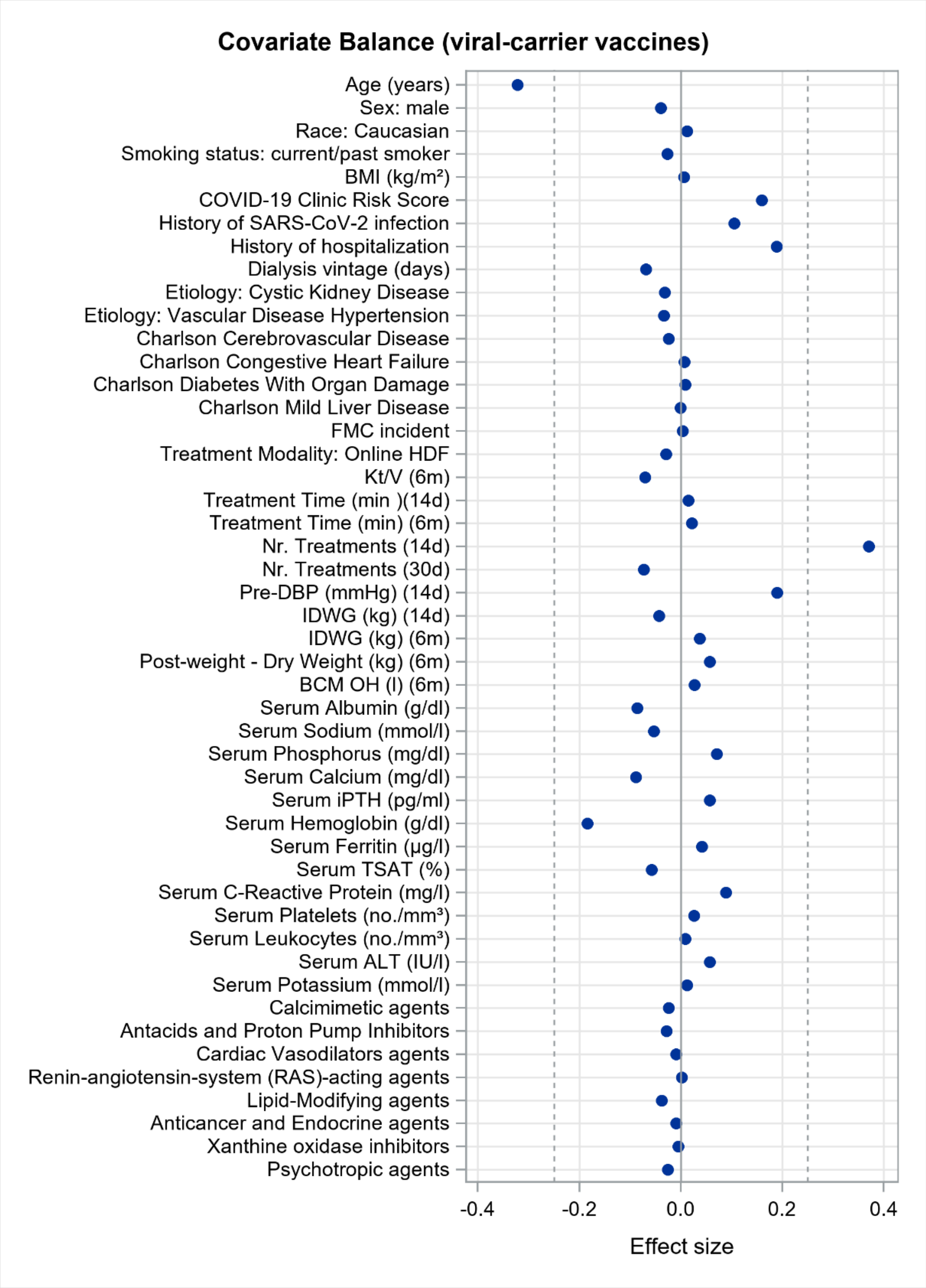

3 Supplementary Table S1 – Covariates included in the outcome risk score model

Table S1. The outcome risk score (ORS) model used to match the vaccinated and unvaccinated patients was assessed with a stepwise logistic regression model (with significance threshold of P-value less than 0.30 for variable entry and 0.15 for variable removal) predicting the SARS-CoV-2 infection in the following 14 days considering the variables listed in the table.

Category	Variable	Reference Time
**Socio-Demographics**		
	Age (years)	at index date
	Sex	
	Race	
	Smoking status	at index date
	Body Mass Index (BMI) (kg/m²)	at index date
	Lean Tissue Index (LTI) (Kg/m²)	average of past 6 months prior to index date
**Etiology**		
	Glomerulonephritis	any time prior to index date
	Diabetic Nephropathy	any time prior to index date
	Cystic Kidney Disease	any time prior to index date
	Chronic Pyelonephritis	any time prior to index date
	Vascular Disease Hypertension	any time prior to index date
	Miscellaneous	any time prior to index date
**Charlson Comorbidity Index Comorbidities** (1)		
	Aids	any time prior to index date
	Cerebrovascular Disease	any time prior to index date
	Chronic Pulmonary Disease	any time prior to index date
	Congestive Heart Failure	any time prior to index date
	Connective Tissue Disorder	any time prior to index date
	Coronary Artery Disease	any time prior to index date
	Dementia	any time prior to index date
	Diabetes with Organ Damage	any time prior to index date
	Diabetes without Complication	any time prior to index date
	Hemiplegia	any time prior to index date
	Metastatic Solid Tumor	any time prior to index date

	Mild Liver Disease	any time prior to index date
	Moderate or Severe Liver Disease	any time prior to index date
	Moderate or Severe Renal Disease	any time prior to index date
	Peptic Ulcer Disease	any time prior to index date
	Peripheral Vascular Disease	any time prior to index date
	Tumor Without Metastasis	any time prior to index date
**COVID-19 related**		
	History of SARS-CoV-2 infection	any time prior to index date
	Local (dialysis center) background risk of SARS-CoV-2 infection calculated from the machine learning enhanced sentinel surveillance system (2)	at index date
**Hospitalizations**		
	History of hospitalization	number within 6 months prior to index date
**Dialysis-related**		
	Dialysis Vintage (days)	at index date
	FMC incident (yes/no): did the patient start the renal replacement therapy not more than 3 months before FMC admission?	
	Treatment Modality	the most used within 30 days prior to index date
	Kt/V	average of past 14 days and 6 months prior to index date
	Treatment Time (min)	average of past 14 days and 6 months prior to index date
	Number of treatments	number within 14 days and 30 days prior to index date
	Access type	the most used within 30 days prior to index date
	Pre-dialysis systolic blood pressure (Pre-SBP) (mmHg)	last and average value of past 14 days prior to index date, average of past 6 months prior to index date
	Pre-dialysis diastolic blood pressure (Pre-DBP) (mmHg)	last and average value of past 14 days prior to index date, average of past 6 months prior to index date

	Interdialytic weight gain (IDWG) (Kg)	last and average value of past 14 days prior to index date, average of past 6 months prior to index date
	Post-Dialysis Weight – Dry weight (Kg)	last and average value of past 14 days prior to index date, average of past 6 months prior to index date
**Body Composition Monitor (BCM)**		
	Overhydration OH (l)	average of past 6 months prior to index date
	relative OH (OH/ECW) (%)	average of past 6 months prior to index date
**Laboratory serum levels**		
	Serum Albumin (g/dl)	last value within 6 months prior to index date
	Serum Sodium (mmol/l)	last value within 6 months prior to index date
	Serum Phosphorus (mg/dl)	last value within 6 months prior to index date
	Serum Calcium (mg/dl)	last value within 6 months prior to index date
	Serum iPTH (pg/ml)	last value within 6 months prior to index date
	Serum Haemoglobin (g/dl)	last value within 6 months prior to index date
	Serum Ferritin (µg/l)	last value within 6 months prior to index date
	Serum Transferrin Saturation (TSAT) (%)	last value within 6 months prior to index date
	Serum C-Reactive Protein (mg/l)	last value within 6 months prior to index date
	Serum Platelets (no./mm^3)	last value within 6 months prior to index date
	Serum Leukocytes (no./mm^3)	last value within 6 months prior to index date
	Serum Alanine Aminotransferase (ALT) (IU/l)	last value within 6 months prior to index date
	Serum Potassium (mmol/l)	last value within 6 months prior to index date
**Medications: occurrence of suggestive ATC codes**		
	Phosphate Binders (V03AE, A12A, A02A, A02A)	active prescription at index date
	Vitamin D and Analogues (H05BX02, A11C)	active prescription at index date
	Calcimimetic agents (H05BX01)	active prescription at index date
	Antacids and Proton Pump Inhibitors (A02B)	active prescription at index date
	Anti-Inflammatory agents (A07E, H02, M01, L04)	active prescription at index date

	Anti-Diabetic Drugs (A10A, A10B, A10X)	active prescription at index date
	Anti-thrombotic agents (B01)	active prescription at index date
	Iron Supplements (B03A)	active prescription at index date
	Erythropoietin (B03X)	active prescription at index date
	Inotropic agents (C01A, C01C)	active prescription at index date
	Anti-Arrhythmic agents (C01B)	active prescription at index date
	Cardiac Vasodilators agents (C01D)	active prescription at index date
	Antihypertensive (C02, C03, C04, C08, C07)	active prescription at index date
	Renin-angiotensin-system (RAS)-acting agents (C09)	active prescription at index date
	Lipid-Modifying agents (C10)	active prescription at index date
	Anticancer and Endocrine agents (L01, L02)	active prescription at index date
	Xanthine Oxidase Inhibitors (M04AA)	active prescription at index date
	Psychotropic agents (N03, N04, N05, N06)	active prescription at index date
	Bronchodilators (R03, R06)	active prescription at index date

4 Supplementary Table S2 – Caliper matching

Table S2. We explored matching with different calipers of the outcome risk score to balance the trade-off between patients' similarity within pairs and sample size. The final caliper used was 0.1.

Caliper	Matched Patients	N per Group	SARS-CoV-2 infection among vaccinated (%)	SARS-CoV-2 infection among controls (%)	P-value
0.001	30510	15255	1.68	3.08	<.0001
0.01	41962	20981	1.82	3.34	<.0001
0.1	44458	22229	1.90	3.43	<.0001
0.2	44718	22359	1.89	3.44	<.0001
1	45090	22545	1.91	3.44	<.0001

5 Supplementary Table S3 – Data management

Table S3. We applied data cleansing to 23 continuous variables considering as missing any data that lied outside the listed upper or lower values. The table S3 also shows the amount of missing data after cleansing procedure. We input missing values, resulting from the above-mentioned data cleansing procedure or native in the data, with the national average calculated on the extracted original dataset (i.e. vaccinated and eligible controls). Before matching, eligible control patients are duplicated on each vaccination date in which they were eligible. BMI, body mass index. LTI, lean tissue index. SBP, systolic blood pressure. DBP, diastolic blood pressure. IDWG, interdialytic weight gain. BCM OH, overhydration by the body composition monitor (BCM; Fresenius). BCM relative OH, relative overhydration by the BCM. TSAT, transferrin saturation.

Variable	Min	Max	Missing (%)
Dialysis Vintage (days)	0	8000	2.6
BMI (kg/m²)	15	50	0.7
LTI (Kg/m²)	7	20	9.0
Kt/V	0.5	3	1.9
Pre-SBP (mmHg)	70	250	0.2
Pre-DBP (mmHg)	40	110	2.2
IDWG (Kg)	-0.6	5.3	2.8
Post-Dialysis Weight – Dry weight (Kg)	-2	4	2.3
BCM OH (l)	-1.5	7	8.7
BCM relative OH (%)	-8	30	9.7
Serum Albumin (g/dl)	2.5	5	1.8
Serum Sodium (mmol/l)	130	150	2.7
Serum Phosphorus (mg/dl)	2	9	3.1
Serum Calcium (mg/dl)	7	11	2.6
Serum iPTH (pg/ml)	15	2000	4.6
Serum Hemoglobin (g/dl)	7	15	2.4
Serum Ferritin (µg/l)	15	2500	4.3
Serum TSAT (%)	0	100	5.1
Serum C-Reactive Protein (mg/l)	0	100	11.2
Serum Platelets (no./mm³)	100	400000	2.4
Serum Leukocytes (no./mm³)	1000	15000	3.0
Serum ALT (IU/L)	0	60	3.1
Serum Potassium (mmol/l)	3	8	1.4

6 Supplementary Table S4 – Vaccinated patients by country and vaccine type

Table S4. Vaccinated patients in the matched sample, by country and type of vaccine.

Country	Vaccine type		Total
	mRNA vaccines	viral-carrier vaccines	
Czech Republic	685	246	**931**
Estonia	111	0	**111**
France	1321	0	**1321**
Croatia	143	0	**143**
Hungary	858	391	**1249**
Italy	1220	0	**1220**
Poland	2243	0	**2243**
Portugal	631	0	**631**
Romania	3454	0	**3454**
Russia	0	1839	**1839**
Slovak Republic	1094	0	**1094**
Slovenia	164	55	**219**
Spain	2071	56	**2127**
Serbia	22	127	**149**
Turkey	38	3730	**3768**
**Total**	**14055**	**6444**	**20499**

7 Supplementary Table S5 - Baseline characteristics of the mRNA vaccines cohort

Table S5. Demographic and clinical characteristics of vaccinated persons and unvaccinated controls at baseline in the sample for mRNA vaccines. Continuous variables are expressed as mean ± standard deviation, categorical variables are expressed as percentage. The mean differences and 95% confidence interval (CI) are related to unvaccinated group minus vaccinated group. For effect size, we used Cohen's d for continuous variables and Cramér's V for categorical ones considering acceptable difference of 0.25 or less. BMI, body mass index. FMC incident, if the patient started the renal replacement therapy not more than 3 months before FMC admission. Online HDF, Online hemodiafiltration. DBP, diastolic blood pressure. IDWG, interdialytic weight gain. BCM OH, overhydration by the body composition monitor (BCM; Fresenius). TSAT, transferrin saturation. ALT, alanine transaminase. 6m, 6 months prior to index date. 14d, past 14 days prior to index date. 30d, past 30 days prior to index date.

Characteristics	Unvaccinated (N=20499)	Vaccinated (N=20499)	Effect Size	Mean Difference [95% CI]
Age (years)	64.7 ± 14.4	67.8 ± 13.8	-0.222	-3.144 [-3.474, -2.813]
Sex: male	59.1	61.2	-0.021	-0.020 [-0.032, -0.009]
Race: Caucasian	60.5	62.7	-0.022	-0.021 [-0.033, -0.010]
Smoking status: current/past smoker	20.9	21.7	-0.009	-0.008 [-0.017, 0.002]
BMI (kg/m²)	27.5 ± 5.8	27.5 ± 5.5	0.007	0.039 [-0.094, 0.171]
Local (dialysis center) background risk of SARS-CoV-2 infection calculated from the machine learning enhanced sentinel surveillance system (2)	0.4 ± 0.4	0.3 ± 0.4	0.092	0.033 [0.025, 0.041]
History of SARS-CoV-2 infection	23.5	22	0.018	0.015 [0.005, 0.025]
History of hospitalization	0.4 ± 0.8	0.3 ± 0.7	0.079	0.062 [0.044, 0.081]
Dialysis vintage (days)	1782.0 ± 1690.2	1838.1 ± 1638.3	-0.034	-56.103 [-95.020, -17.186]
Etiology: Cystic Kidney Disease	6.9	6.8	0.003	0.002 [-0.004, 0.008]
Etiology: Vascular Disease Hypertension	10.2	11.1	-0.015	-0.009 [-0.017, -0.002]
Charlson Cerebrovascular Disease	14.3	14.8	-0.006	-0.004 [-0.013, 0.004]
Charlson Congestive Heart Failure	25.2	26.1	-0.010	-0.009 [-0.019, 0.001]
Charlson Diabetes with Organ Damage	27	28.5	-0.016	-0.015 [-0.025, -0.004]
Charlson Mild Liver Disease	11.4	10.9	0.008	0.005 [-0.002, 0.012]

FMC incident	84	84.4	-0.006	-0.004 [-0.013, 0.004]
Treatment Modality: Online HDF	53	56.2	-0.032	-0.032 [-0.043, -0.020]
Kt/V (6m)	1.6 ± 0.4	1.7 ± 0.4	-0.097	-0.036 [-0.044, -0.027]
Treatment Time (min) (14d)	239.2 ± 21.4	241.5 ± 15.3	-0.124	-2.306 [-2.741, -1.871]
Treatment Time (min) (6m)	238.1 ± 21.0	240.5 ± 15.3	-0.128	-2.354 [-2.783, -1.924]
Nr. Treatments (14d)	6.6 ± 1.3	6.6 ± 0.8	0.022	0.024 [-0.001, 0.050]
Nr. Treatments (30d)	12.6 ± 3.0	13.2 ± 1.6	-0.247	-0.599 [-0.655, -0.542]
Pre-DBP (mmHg) (14d)	72.4 ± 12.4	70.8 ± 12.1	0.127	1.555 [1.268, 1.841]
IDWG (kg) (14d)	2.1 ± 1.1	2.0 ± 1.0	0.079	0.082 [0.058, 0.107]
IDWG (kg) (6m)	2.0 ± 0.8	2.0 ± 0.8	0.021	0.017 [-0.002, 0.036]
Post-weight - Dry Weight (kg) (6m)	0.3 ± 0.6	0.3 ± 0.5	0.037	0.021 [0.008, 0.035]
BCM OH (l) (6m)	1.8 ± 1.4	1.8 ± 1.4	0.035	0.049 [0.017, 0.082]
Serum Albumin (g/dl)	3.9 ± 0.4	3.9 ± 0.4	-0.064	-0.024 [-0.033, -0.015]
Serum Sodium (mmol/l)	138.2 ± 3.1	138.2 ± 3.1	-0.015	-0.047 [-0.120, 0.025]
Serum Phosphorus (mg/dl)	4.7 ± 1.3	4.6 ± 1.3	0.069	0.090 [0.060, 0.121]
Serum Calcium (mg/dl)	8.9 ± 0.7	8.9 ± 0.7	-0.069	-0.046 [-0.062, -0.030]
Serum iPTH (pg/ml)	358.8 ± 310.7	329.0 ± 288.5	0.100	29.855 [22.845, 36.865]
Serum Hemoglobin (g/dl)	11.0 ± 1.3	11.2 ± 1.2	-0.134	-0.172 [-0.202, -0.142]
Serum Ferritin (µg/l)	590.6 ± 430.4	589.0 ± 426.7	0.004	1.607 [-8.413, 11.627]
Serum TSAT (%)	29.5 ± 14.3	30.0 ± 14.4	-0.038	-0.546 [-0.882, -0.211]
Serum C-Reactive Protein (mg/l)	11.3 ± 14.3	10.4 ± 13.0	0.071	0.966 [0.646, 1.285]
Serum Platelets (no./mm^3)	190701.4 ± 72546.0	190027.8 ± 67978.0	0.010	673.573 [-970.103, 2317.249]
Serum Leukocytes (no./mm^3)	6685.3 ± 2059.3	6596.1 ± 1979.5	0.044	89.236 [42.010, 136.462]
Serum ALT (IU/l)	15.7 ± 8.5	15.4 ± 8.3	0.030	0.251 [0.054, 0.448]
Serum Potassium (mmol/l)	4.9 ± 0.7	4.9 ± 0.7	-0.009	-0.007 [-0.024, 0.011]
Calcimimetic agents	12.2	12.6	-0.006	-0.004 [-0.012, 0.004]
Antacids and Proton Pump Inhibitors	45.9	47.5	-0.017	-0.017 [-0.028, -0.005]
Cardiac Vasodilators agents	7.7	8.4	-0.013	-0.007 [-0.013, -0.001]
Renin-angiotensin-system (RAS)-acting agents	28.7	28.8	-0.001	-0.001 [-0.011, 0.010]

Lipid-Modifying agents	36.5	40	-0.036	-0.035 [-0.046, -0.024]
Anticancer and Endocrine agents	1.2	1.3	-0.004	-0.001 [-0.003, 0.002]
Xanthine oxidase inhibitors	19	19.8	-0.010	-0.008 [-0.017, 0.002]
Psychotropic agents	27.3	29	-0.019	-0.017 [-0.028, -0.007]

8 Supplementary Table S6 - Baseline characteristics of the viral-carrier vaccines cohort

Table S6. Demographic and clinical characteristics of vaccinated persons and unvaccinated controls at baseline in the sample for viral-carrier vaccines. Continuous variables are expressed as mean ± standard deviation, categorical variables are expressed as percentage. The mean differences and 95% confidence interval (CI) are related to unvaccinated group minus vaccinated group. For effect size, we used Cohen's d for continuous variables and Cramér's V for categorical ones considering acceptable difference of 0.25 or less. BMI, body mass index. FMC incident, if the patient started the renal replacement therapy not more than 3 months before FMC admission. Online HDF, Online hemodiafiltration. DBP, diastolic blood pressure. IDWG, interdialytic weight gain. BCM OH, overhydration by the body composition monitor (BCM; Fresenius). TSAT, transferrin saturation. ALT, alanine transaminase. 6m, 6 months prior to index date. 14d, past 14 days prior to index date. 30d, past 30 days prior to index date.

Characteristics	Unvaccinated (N=6444)	Vaccinated (N=6444)	Effect Size	Mean Difference [95% CI]
Age (years)	58.5 ± 13.9	62.9 ± 13.1	-0.322	-4.351 [-4.819, -3.884]
Sex: male	56.4	60.3	-0.040	-0.039 [-0.056, -0.022]
Race: Caucasian	50.1	48.8	0.012	0.012 [-0.005, 0.030]
Smoking status: current/past smoker	26.7	29.1	-0.026	-0.024 [-0.039, -0.008]
BMI (kg/m²)	27.4 ± 5.7	27.3 ± 5.5	0.006	0.036 [-0.157, 0.230]
Local (dialysis center) background risk of SARS-CoV-2 infection calculated from the machine learning enhanced sentinel surveillance system (2)	0.5 ± 0.4	0.4 ± 0.4	0.160	0.059 [0.047, 0.072]
History of SARS-CoV-2 infection	14.5	7.8	0.106	0.067 [0.056, 0.077]
History of hospitalization	0.3 ± 0.7	0.2 ± 0.6	0.189	0.121 [0.099, 0.143]
Dialysis vintage (days)	2062.3 ± 1780.3	2183.3 ± 1762.6	-0.068	-121.012 [-182.185, -59.838]
Etiology: Cystic Kidney Disease	5.1	6.6	-0.032	-0.015 [-0.023, -0.007]
Etiology: Vascular Disease Hypertension	11.3	13.5	-0.033	-0.022 [-0.033, -0.011]
Charlson Cerebrovascular Disease	13.3	14.9	-0.024	-0.017 [-0.029, -0.005]
Charlson Congestive Heart Failure	23	22.5	0.007	0.006 [-0.009, 0.020]
Charlson Diabetes with Organ Damage	26.4	25.6	0.009	0.008 [-0.007, 0.023]

Charlson Mild Liver Disease	11.5	11.6	-0.001	-0.000 [-0.012, 0.011]
FMC incident	66.4	66	0.004	0.004 [-0.013, 0.020]
Treatment Modality: Online HDF	49.6	52.5	-0.029	-0.029 [-0.046, -0.011]
Kt/V (6m)	1.7 ± 0.3	1.7 ± 0.3	-0.071	-0.020 [-0.029, -0.010]
Treatment Time (min) (14d)	255.7 ± 46.6	255.0 ± 42.9	0.015	0.656 [-0.891, 2.203]
Treatment Time (min) (6m)	255.1 ± 45.8	254.2 ± 40.9	0.021	0.930 [-0.569, 2.429]
Nr. Treatments (14d)	6.6 ± 1.3	6.2 ± 0.9	0.371	0.398 [0.361, 0.435]
Nr. Treatments (30d)	12.6 ± 2.7	12.8 ± 1.7	-0.073	-0.167 [-0.245, -0.088]
Pre-DBP (mmHg) (14d)	74.1 ± 11.8	71.9 ± 11.8	0.189	2.236 [1.829, 2.644]
IDWG (kg) (14d)	2.2 ± 1.1	2.3 ± 1.1	-0.043	-0.047 [-0.084, -0.009]
IDWG (kg) (6m)	2.3 ± 0.9	2.2 ± 0.8	0.037	0.032 [0.002, 0.062]
Post-weight - Dry Weight (kg) (6m)	0.3 ± 0.7	0.3 ± 0.6	0.057	0.035 [0.014, 0.057]
BCM OH (l) (6m)	1.7 ± 1.4	1.6 ± 1.3	0.027	0.037 [-0.010, 0.084]
Serum Albumin (g/dl)	3.9 ± 0.4	4.0 ± 0.3	-0.086	-0.031 [-0.043, -0.018]
Serum Sodium (mmol/l)	138.7 ± 3.1	138.9 ± 3.1	-0.053	-0.165 [-0.271, -0.058]
Serum Phosphorus (mg/dl)	4.8 ± 1.3	4.7 ± 1.2	0.071	0.088 [0.046, 0.131]
Serum Calcium (mg/dl)	8.8 ± 0.7	8.9 ± 0.7	-0.089	-0.062 [-0.086, -0.038]
Serum iPTH (pg/ml)	394.9 ± 331.4	376.4 ± 316.3	0.057	18.457 [7.271, 29.643]
Serum Hemoglobin (g/dl)	11.2 ± 1.5	11.5 ± 1.4	-0.184	-0.265 [-0.315, -0.215]
Serum Ferritin (µg/l)	673.5 ± 500.1	653.3 ± 475.5	0.041	20.150 [3.299, 37.000]
Serum TSAT (%)	35.0 ± 18.6	36.1 ± 18.7	-0.058	-1.080 [-1.724, -0.436]
Serum C-Reactive Protein (mg/l)	12.6 ± 16.2	11.3 ± 14.6	0.089	1.377 [0.844, 1.909]
Serum Platelets (no./mm³)	204859.1 ± 63749.0	203244.5 ± 61396.5	0.026	1614.678 [-546.488, 3775.844]
Serum Leukocytes (no./mm³)	6692.0 ± 2105.4	6673.8 ± 2018.3	0.009	18.242 [-52.974, 89.457]
Serum ALT (IU/l)	13.4 ± 8.3	12.9 ± 7.7	0.057	0.457 [0.180, 0.734]
Serum Potassium (mmol/l)	5.0 ± 0.7	5.0 ± 0.7	0.012	0.008 [-0.015, 0.032]
Calcimimetic agents	6.5	7.7	-0.024	-0.012 [-0.021, -0.003]
Antacids and Proton Pump Inhibitors	29.2	31.8	-0.028	-0.026 [-0.042, -0.010]
Cardiac Vasodilators agents	4.3	4.7	-0.009	-0.004 [-0.011, 0.003]
Renin-angiotensin-system (RAS)-acting agents	22.4	22.2	0.002	0.002 [-0.013, 0.016]

Lipid-Modifying agents	12.5	15.1	-0.038	-0.026 [-0.038, -0.014]
Anticancer and Endocrine agents	0.6	0.8	-0.009	-0.002 [-0.004, 0.001]
Xanthine oxidase inhibitors	7.5	7.8	-0.005	-0.002 [-0.012, 0.007]
Psychotropic agents	12.1	13.8	-0.025	-0.017 [-0.029, -0.005]

9 Supplementary Table S7 – No. At Risk and Events in the mRNA vaccines cohort

Table S7. Number of patients at risk at each time point and cumulative number of events are shown for each outcome: SARS-CoV-2 infection (up) and COVID-19 related death (down).

**mRNA vaccine cohort: documented SARS-CoV-2 infection**								
**Follow-Up (days)**	**0**	**15**	**30**	**45**	**60**	**75**	**90**	**Residual No. of Events**
**No. at Risk**								
ALL	28110	18602	13771	10466	7813	6317	4796	
Unvaccinated	14055	9249	6741	5016	3666	2911	2188	
Vaccinated	14055	9353	7030	5450	4147	3406	2608	
**Cumulative No. of Events**								
ALL	0	382	568	683	753	811	829	21
Unvaccinated	0	234	341	425	485	535	546	16
Vaccinated	0	148	227	258	268	276	283	5
**mRNA vaccine cohort: COVID-19 related death**								
**Follow-Up (days)**	**0**	**15**	**30**	**45**	**60**	**75**	**90**	**Residual No. of Events**
**No. at Risk**								
ALL	28110	18933	14199	10922	8267	6733	5129	
Unvaccinated	14055	9463	7027	5346	4009	3232	2450	
Vaccinated	14055	9470	7172	5576	4258	3501	2679	
**Cumulative No. of Events**								
ALL	0	29	89	124	155	179	186	15
Unvaccinated	0	20	53	78	104	124	131	14
Vaccinated	0	9	36	46	51	55	55	1

10 Supplementary Table S8 – No. At Risk and Events in the viral-carrier vaccines cohort

Table S8. Number of patients at risk at each time point and cumulative number of events are shown for each outcome: SARS-CoV-2 infection (up) and COVID-19 related death (down).

**Viral carrier vaccine cohort: documented SARS-CoV-2 infection**								
**Follow-Up (days)**	**0**	**15**	**30**	**45**	**60**	**75**	**90**	**Residual No. of Events**
**No. at Risk**								
ALL	12888	10302	8615	6496	4201	2676	1728	
Unvaccinated	6444	5122	4268	3172	2011	1253	804	
Vaccinated	6444	5180	4347	3324	2190	1423	924	
**Cumulative No. of Events**								
ALL	0	95	167	221	258	282	293	4
Unvaccinated	0	61	96	136	161	174	178	3
Vaccinated	0	34	71	85	97	108	115	1
**Viral carrier vaccine cohort: COVID-19 related death**								
**Follow-Up (days)**	**0**	**15**	**30**	**45**	**60**	**75**	**90**	**Residual No. of Events**
**No. at Risk**								
ALL	12888	10390	8751	6646	4351	2792	1808	
Unvaccinated	6444	5177	4350	3273	2122	1341	859	
Vaccinated	6444	5213	4401	3373	2229	1451	949	
**Cumulative No. of Events**								
ALL	0	5	17	39	47	57	61	3
Unvaccinated	0	5	12	30	38	47	51	3
Vaccinated	0	0	5	9	9	10	10	0

11 Supplementary Table S9 – Cumulative patients with 2 doses of COVID-19 vaccine

Table S9. Cumulative patients with two doses administered in their follow-up period by vaccine type.

**Cumulative patients with two doses of COVID-19 vaccine N (%)**				
**Follow-Up (days)**	**0**	**30**	**60**	**90**
mRNA vaccine	0 (0.0)	6308 (44.9)	7445 (53.0)	7465 (53.1)
Viral carrier vaccine *	0 (0.0)	3109 (48.2)	3913 (60.7)	3991 (61.9)

* The viral-carrier vaccines considered in our study were mostly administered in Eastern Europe countries. In those countries, only Sinovac-CoronaVac and Sputnik V COVID-19 vaccines were available. According to protocols, second doses for both these viral-carrier vaccines should be administered at 2-4 weeks and 3 weeks respectively.

12 References

1. Charlson ME, Pompei P, Ales KL, MacKenzie CR. A new method of classifying prognostic comorbidity in longitudinal studies: Development and validation. J Chronic Dis [Internet]. 1987 Jan;40(5):373–83. Doi: 10.1016/0021-9681(87)90171-8

2. Bellocchio F, Carioni P, Garbelli M, Martínez-Martínez F, Larkin JW, Usvyat LA, et al. Enhanced sentinel surveillance system for COVID-19 outbreak prediction in a European dialysis clinics network. J Am Soc Nephrol [Internet]. 2020;31:275.
